# Modeling epigenetic modifications in renal development and disease with organoids and genome editing

**DOI:** 10.1242/dmm.035048

**Published:** 2018-11-20

**Authors:** Carmen Hurtado del Pozo, Elena Garreta, Juan Carlos Izpisúa Belmonte, Nuria Montserrat

**Affiliations:** 1 Pluripotency for organ regeneration. Institute for Bioengineering of Catalonia (IBEC), the Barcelona Institute of Technology (BIST), 08028 Barcelona, Spain; 2 Gene Expression Laboratory, Salk Institute for Biological Studies, La Jolla, CA 92037, USA

**Keywords:** Epigenetics, Genome editing, Organoids, CRISPR/Cas9

## Abstract

Understanding epigenetic mechanisms is crucial to our comprehension of gene regulation in development and disease. In the past decades, different studies have shown the role of epigenetic modifications and modifiers in renal disease, especially during its progression towards chronic and end-stage renal disease. Thus, the identification of genetic variation associated with chronic kidney disease has resulted in better clinical management of patients. Despite the importance of these findings, the translation of genotype–phenotype data into gene-based medicine in chronic kidney disease populations still lacks faithful cellular or animal models that recapitulate the key aspects of the human kidney. The latest advances in the field of stem cells have shown that it is possible to emulate kidney development and function with organoids derived from human pluripotent stem cells. These have successfully recapitulated not only kidney differentiation, but also the specific phenotypical traits related to kidney function. The combination of this methodology with CRISPR/Cas9 genome editing has already helped researchers to model different genetic kidney disorders. Nowadays, CRISPR/Cas9-based approaches also allow epigenetic modifications, and thus represent an unprecedented tool for the screening of genetic variants, epigenetic modifications or even changes in chromatin structure that are altered in renal disease. In this Review, we discuss these technical advances in kidney modeling, and offer an overview of the role of epigenetic regulation in kidney development and disease.

## Abstract (precis)

**Summary:** In this Review, we provide an overview on how epigenetic processes are altered in kidney development and disease, and discuss how CRISPR-modified kidney organoids can help us to understand the function of epigenetic marks.

## Introduction

Epigenetics have traditionally been linked with developmental biology. In fact, the first definition of epigenetics came from a developmental biologist, Conrad Hal Waddington, who defined the term in the early 1940s. Since this first definition, the concept and role of epigenetics has evolved. Nowadays, we understand epigenetics as regulatory mechanisms that stabilize gene expression patterns without altering the DNA sequence. Rather, these are mediated by heritable chemical modifications to DNA and histones (Box 1, ‘Glossary’) that affect DNA accessibility and modulate chromatin structure.

**Box 1. Glossary**

**Advanced glycation end products (AGEs):** proteins or lipids that become glycated (sometimes also referred to as non-enzymatically glycosylated) by the covalent bonding of glucose or fructose as a result of exposure to sugars.

**Autosomal dominant polycystic kidney disease (ADPKD):** an inherited condition that causes small, fluid-filled sacs called cysts to develop in the kidneys. Although children affected by ADPKD are born with the condition, it rarely causes any noticeable problems until the cysts grow large enough to affect the kidneys' functions. In most cases, this does not occur until a person is between 30 and 60 years of age.

**Chronic kidney disease (CKD):** a kidney condition in which there is a gradual loss of kidney function over a period of months or years. Specifically, it implies structural or functional abnormalities of the kidney that induce decreased glomerular filtration rates and can lead to kidney failure.

**Chromatin immunoprecipitation-DNA sequencing (ChIP-Seq):** a method used to analyze protein interactions with DNA. It combines chromatin immunoprecipitation (ChIP) with massively parallel DNA sequencing to identify the binding sites of DNA-associated proteins.

**Clear cell renal cell carcinoma (ccRCC):** the major subtype of kidney cancer. It is characterized by frequent inactivation of *VHL*, which can be found in 80–90% of the cases.

**Collecting duct system:** tubules and ducts that physically connect nephrons to a minor calyx or directly to the renal pelvis.

**Congenital abnormalities of the kidney and urinary tract (CAKUT):** common congenital abnormalities, occurring at a frequency of ∼1 in 500 fetal ultrasound examinations. The molecular pathogenesis of the disease remains unclear, although numerous studies support the influence of epigenetic and environmental factors on kidney development and the natural history of CAKUT, suggesting that the pathogenesis of this syndrome is multifactorial.

**CpG island:** regions >0.5 Kb with a high concentration of cytosine (C) and guanine (G) (≥55%). CpG islands are sites of transcription initiation, including those that are distant from the currently annotated promoters. CpG silencing in promoter regions is achieved through dense CpG methylation or Polycomb recruitment.

**Crotonylation:** a post-translational modification of lysine residues on histones by the addition of crotonyl (the CH3-CH=CH-CO- radical derived from crotonic acid) groups.

**CRISPR/Cas9 technology:** a genome editing system that uses the RNA-guided CRISPR-associated endonuclease Cas9 and a single-guide RNA (sgRNA) to induce a DNA double-strand break (DSB) at a specific DNA region of interest. This site specificity is determined by sequence complementarity with the sgRNA. The DSB can then be repaired through different pathways, leading to the creation small insertion and deletion mutations, or to the introduction of precise nucleotide alterations when exogenous homologous DNA templates are provided.

**Diabetic nephropathy (DN):** a syndrome of albuminuria, declining glomerular filtration rate, arterial hypertension and increased cardiovascular risk that affects 25–40% of type 1 (insulin-dependent) and type 2 (non-insulin-dependent) diabetic patients. Diabetic nephropathy is the leading cause of end-stage renal disease (ESRD), present in ∼25–40% of patients with long-term diabetes worldwide.

**Dicer1:** an enzyme that is part of the RNase III family. Dicer1 cleaves double-stranded RNA (dsRNA) and pre-microRNA (pre-miRNA) into microRNA. Dicer1 facilitates the activation of the RNA-induced silencing complex (RISC), which is essential for RNA interference.

**Doxycycline:** a tetracycline-like antibiotic. In genetic engineering, doxycycline is used as the regulator for inducible gene expression systems, whereby expression of an engineered gene from a tetracycline-dependent promoter depends on either the presence (Tet-On) or absence (Tet-Off) of doxycycline.

**Drosha:** the core nuclease that executes the initiation step of miRNA processing in the nucleus.

**Embryoid bodies (EBs):** 3D cell aggregates that are generated *in vitro* by suspension culture of pluripotent stem cells. During the first 3 days in suspension culture, EBs can mirror the development of the three germ layers – the endoderm, mesoderm and ectoderm – of the mammalian embryo.

**End-stage renal disease (ESRD):** kidney disease characterized by gradual loss of kidney function. In early stages, the disease is asymptomatic. Once the symptoms appear, the damage is irreversible.

**Focal segmental glomerulosclerosis (FSGS):** a cause of nephrotic syndrome in children and adolescents, as well as a leading cause of kidney failure in adults. It is characterized by generalized edema, massive proteinuria, hypoalbuminemia and hyperlipidemia.

**Genomic imprinting:** an epigenetic phenomenon that causes genes to be expressed in a parent-of-origin-specific manner. It involves DNA methylation and histone methylation. These epigenetic signatures are maintained through mitotic cell divisions in the somatic cells of an organism.

**Genome-wide association studies (GWAS):** observational study of a genome-wide set of genetic variants in different individuals to assess if any variant is associated with a phenotypic trait. GWAS typically focus on associations between single-nucleotide polymorphisms (SNPs) and traits like major human diseases, but can equally be applied to any other organism or phenotype.

**Glomerular filtration rate (GFR):** the flow rate of filtered fluid through the kidney.

**Glycolysis:** the metabolic pathway that converts glucose into pyruvate. The energy released in this process is used to form the high-energy molecules adenosine triphosphate (ATP) and reduced nicotinamide adenine dinucleotide (NADH).

**Hemoglobin A1c (HbA1c):** glycated HbA1c is a common indicator used in clinic to measure the average plasma glucose concentration in a patient. It is the product of an irreversible reaction in which a molecule of glucose attaches to the N-terminal valine of the hemoglobin β-chain. This irreversible reaction, together with the life span of erythrocytes (∼120 days) make HbA1c a good marker to monitor long-term glycemia.

**Histones:** a family of basic proteins that associate with the DNA in the nucleus and compact it into chromatin.

**Histone crotonylation:** post-translational modification of lysine residues in a histone by the introduction of crotonyl groups.

**Hypoxia:** a condition of insufficient oxygen supply.

***HOTAIR*:** a gene located within the Homeobox C (*HOXC*) gene cluster on chromosome 12 and is co-expressed with the HOXC genes. It functions through an RNA product that binds lysine specific demethylase 1 (LSD1) and Polycomb repressive complex 2 (PRC2), and serves as a scaffold to assemble these regulators at the *HOXD* gene cluster, thereby promoting epigenetic repression of *HOXD*. It was the first example of an RNA expressed on one chromosome that has been found to influence transcription on another chromosome.

**Intermediate mesoderm (IM):** the mesodermal lineage that is specified early in development between the paraxial and lateral plate mesoderm. Later in development, it gives rise to the urogenital structures, including the definitive kidney.

**Lin28A:** an RNA-binding protein that binds to and enhances the translation of the insulin-like growth factor 2 (*IGF-2*) mRNA. Lin28 binds to the let-7 pre-miRNA and blocks production of the mature let-7 miRNA in mouse embryonic stem cells.

**Long non-coding RNAs (lncRNAs):** transcribed RNA molecules with a length of more than 200 nucleotides that do not encode proteins.

**Mesenchymal-to-epithelial transition (MET):** a reversible biological process that involves the transition from spindle-shaped mesenchymal cells to planar arrays of polarized cells called epithelia.

**Metanephric mesenchyme (MM):** the embryonic cell lineage that arises from the posterior part of the intermediate mesoderm. During development, a subset of cells of the MM condenses around each ureteric bud (UB) tip to form the cap mesenchyme (CM), the multipotent population of progenitors that will develop into the nephrons, whereas the UB will constitute the collecting duct system. All the different cells present in the mature nephron derive from a population of Six2^+^ renal progenitor cells that reside in CM (Kobayashi et al., 2008).

**MicroRNAs (miRNAs):** small non-coding RNA molecules (containing ∼22 nucleotides) that function in RNA silencing and post-transcriptional regulation of gene expression.

**Notch pathway:** a highly conserved cell signaling system present in most multicellular organisms. Notch proteins play an essential role in developmental patterning, determining lateral inhibition and lateral induction (Andersson et al., 2011).

**Nephron:** the structural and functional unit of the kidney. It is composed of a renal corpuscle and a renal tubule. A healthy adult has 0.8 to 1.5 million nephrons in each kidney.

**Oxidative phosphorylation (OXPHOS):** the metabolic pathway in which cells oxidize nutrients in order to release energy. ATP is formed as a result of the transfer of electrons to oxygen by a series of electron carriers. This process, which takes place in mitochondria, is the major source of ATP in aerobic organisms.

**Hox genes:** a group of transcription factors that control the body plan of an embryo along the head-tail axis. Hox proteins define the types of appendages (like legs, antennae, and wings in fruit flies) or the different types of vertebrae (in humans) that will form on a segment.

**Safe harbor locus (AAVS1):** a genomic locus that serves as an ideal site for the integration of a transgene without causing harm and with a consistent level of expression. In humans, there is a safe-harbor site on chromosome 19 (locus PPP1R12C) called AAVS1.

**TALEN:** transcription activator-like effector nuclease. A genome-editing nuclease that can be engineered to bind and cleave any desired DNA sequence.

**Tiling arrays:** a subtype of microarray chips. They are similar to traditional gene expression microarrays, but they contain probes for functionally uncharacterized regions through the genome. This technique is useful for characterizing regions that are sequenced, but the local functions are largely unknown.

**Trithorax/Polycomb:** a group of proteins that regulate gene expression in an opposite manner through development. The main action of Trithorax proteins is to maintain gene expression, while Polycomb proteins have a repressive function. Polycomb proteins are well known for silencing Hox genes in *Drosophila melanogaster* and regulate homeotic gene regulation and X chromosome inactivation in mammals. Trithorax proteins are also necessary for X chromosome inactivation and activation of Hox genes. Both groups of proteins regulate gene expression by modulating the chromatin structure.

**Ureteric bud (UB):** a protrusion from the mesonephric duct that forms during the development of the urinary organs. It later develops into a conduit for urine drainage from the kidneys.

**Wnt signaling:** a group of signal transduction pathways with key functions in embryonic development. Three Wnt signaling pathways have been characterized: the canonical Wnt pathway, the non-canonical planar cell polarity pathway and the non-canonical Wnt/calcium pathway. All three pathways are activated by binding a Wnt protein ligand to a Frizzled family receptor, which passes the biological signal to the Dishevelled protein inside the cell. They are highly evolutionarily conserved in animals, from fruit flies to humans.

**X chromosome inactivation:** a process by which one of the copies of the X chromosome present in female mammals is inactivated. The inactive X chromosome is silenced by it being packaged in such a way that it has a transcriptionally inactive structure.

Epigenetic modifications include DNA methylation, post-translational modifications of histones, and long non-coding RNAs (lncRNAs; Box 1; Box 2, ‘Epigenetic regulatory mechanisms’). These three mechanisms are responsible for tissue-specific gene expression during growth and are the key regulators of important development events, including X chromosome inactivation (Box 1) (Lyon, 1961), genomic imprinting (Box 1) (Li et al., 1993; Reik et al., 1987) and patterning by Hox genes (Box 1) (McGinnis and Krumlauf, 1992). Histone methylation, hypermethylation of CpG islands (Box 1) in DNA, and expression of lncRNAs such as *HOTAIR* (Box 1), and of the Trithorax (TrxG) and Polycomb (PcG; also known as Pcgf) gene families (Box 1), act at several layers of the chromatin structure, compartmentalizing the genome into active and inactive domains to guarantee the correct differentiation into tissue-specific cell types (Kiefer, 2007). Although our knowledge of the epigenetics of development has increased (Theunissen and Jaenisch, 2017), our understanding of the global epigenetic states of lineage-committed progenitors during organogenesis, specifically in kidney, remains limited.

**Box 2. Epigenetic regulatory mechanisms**

Epigenetics was defined for the first time in 1942 by the developmental biologist Conrad H. Waddington as a mode of inheritance of traits not encoded in the DNA sequence (Waddington, 2012). Currently, epigenetics can be defined as the collection of mechanisms involved in regulating gene expression without affecting the DNA sequence (Berger et al., 2009). Epigenetic mechanisms are mediated by chemical modifications to the chromatin: DNA itself and histones. These modifications are heritable, leading to the stable propagation of phenotype changes, a concept known as epigenetic memory. Epigenetic modifications include cytosine methylation of DNA (DNA methylation) (Avery et al., 1944; McCarty and Avery, 1946), post-translational modifications of histones (Allfrey et al., 1964) and non-coding RNAs (Lee et al., 1993; Wightman et al., 1993). In recent decades, the identification of these epigenetic traits has emerged as a potential selection criterion in personalized medicine because, unlike DNA sequence variations, epigenetic modifications are potentially reversible.

**DNA methylation**

DNA methylation is the most frequently studied epigenetic modification involved in regulating transcriptional activity. It consists in the covalent addition of a methyl group to the cytosine (C) nucleotide, giving rise to 5-methyl-cytosine (5-mC). In humans, *de novo* DNA methylation is catalyzed by the DNA methyl transferases (DNMTs) 3A and 3B, while DNMT1 maintains DNA methylation through cell divisions. Gene expression activation is often associated with demethylation/hypomethylation of promoter and enhancer sequences, which can be achieved through inhibition of DNMT1 or through oxidation of 5-mC into 5-hydroxymethylcytosine (5-hmC) by the ten-eleven-translocation (TET) proteins (TET1–3). 5-hmC is then converted to unmodified cytosine by DNA-replication-dependent dilution or by glycosylase-initiated base excision. So far, a growing number of studies have correlated aberrant DNA methylation with human disease. In addition, DNA methylation regulates important processes, including imprinting (Li et al., 1993), cell differentiation (Khavari et al., 2010) and X chromosome inactivation (Kaslow and Migeon, 1987).

**Post-translational modifications of histones**

Histones are a family of small basic proteins that envelop DNA, forming an efficient DNA-packaging unit called nucleosome, which is the basic structural and functional unit of chromatin. Post-translational modifications (PTMs) of histones occur at their amino-terminal ends (histone tails), mainly on specific lysine (K) and arginine (R) residues, and can affect DNA availability for transcription factor binding, thereby regulating gene expression via changes in chromatin structure. The combination of different histone marks, or the histone code, in genomic regulatory regions, such as enhancers and promoters, induces either transcriptionally permissive (euchromatin) or repressive (heterochromatin) chromatin states. In general, histone PTM-mediated chromatin organization affects complex biological processes, including gene expression (Wu et al., 1986), DNA repair (Huang et al., 2018) and chromosome condensation (Bannister and Kouzarides, 2011). Histone PTMs include methylation, acetylation, phosphorylation, sumoylation, crotonylation and ubiquitination. Among these, sumoylation and ubiquitination are less understood. The enzymes that deposit, recognize and remove PTMs from histones are often referred to as writers, readers and erasers of the histone code, respectively. The classification of enzymes involved in histone PTMs during kidney development, together with the target genes these PTMs affect, are listed in Table 1.

Histone methyltransferases (HMTs) add methyl groups to histones, while histone lysine demethylases (HDMs) remove them. HMTs and HDMs are positive or negative regulators of gene transcription, depending on the amino acid, histone tail position or the number of methyl groups in the PTM (mono-, di- or trimethyl). In general, histone demethylation is quite dynamic, whereas methylation is more unvarying and linked to long-term cellular epigenetic memory. Histone lysine methylation (HKme) can be associated with either active or inactive promoters, depending on the methylated lysine (K). In general, trimethylation at H3K9 (H3K9me3), H3K27 (H3K27me3) and H4K20 (H4K20me3) correlates with inactive genes, whereas trimethylation at H3K4 (H3K4me3) and H3K36 (H3K36me3) correlates with promoters of actively transcribed genes.

Histone acetylation involves the addition of acetyl groups tovarious histones and is mediated by histone acetyl transferases (HATs). The reverse process, histone deacetylation, is catalyzed by histone deacetylases (HDACs). Histone lysine acetylation (HKac) marks – such as H3K9ac, H3K14ac, and H4Kac – are associated with active promoters. Furthermore, enzymes like kinases and phosphatases can also regulate the phosphorylation status of histones. Phosphorylation of H2A has proved to play a major role in DNA damage response (Goldknopf et al., 1975).

Histone phosphorylation can occur in all four nucleosomal histone tails. These possess acceptor sites that can be phosphorylated by a number of protein kinases (at serine, threonine and tyrosine residues) and dephosphorylated by phosphatases. So far, a large number of phosphorylated residues on histones have been described. The best-known function of histone phosphorylation takes place during cellular response to DNA damage, when phosphorylated histone H2A(X) demarcates chromatin domains around the site of DNA damage (break). Nowadays the identification of new phosphorylation sites and characterization is still under investigation. The current knowledge on histone phosphorylation, their regulation and function in modulation of chromatin structure and gene expression has been extensively reviewed elsewhere (Rossetto et al., 2012).

Histone sumoylation is a histone modification directed by an enzymatic cascade in which small ubiquitin-like modifier (SUMO) proteins are attached to, or detached from, other proteins to change their function in cells. Different works have revealed a general causal relationship between sumoylation and gene repression. However, the dual role of SUMO in gene regulation is demonstrated by the observations that sumoylation of certain transcription factors, including Ikaros (also known as Ikzf1), enhances transcriptional activity. On the other hand, inducing hypersumoylation by targeting SUMO and/or Ubc9 to specific gene promoters primarily induces gene repression (Chupreta et al., 2005; Nathan et al., 2003). Conversely, overexpressing SUMO isopeptidases or depleting cells of Ubc9 or SUMO enhances ectopic gene expression (Ouyang et al., 2009; Poulin et al., 2005).

Histone crotonylation was described for the first time in 2011 (Tan et al., 2011), but little is known about its regulation and tissue-specific functions. What is known is that it is a post-translational modification of lysine residues by the introduction of crotonyl groups. It takes place in most of all core histones and marks either active promoters or potential enhancers. *In vitro*, histone crotonylation by p300 has been shown to promote transcription, and increasing the cellular concentration of crotonyl-CoA affects gene expression (Sabari et al., 2015). *In vivo*, it has recently been published that histone H3 crotonylation at lysine 18 is an abundant modification in the small intestine crypt and colon in mice, and is linked to gene regulation (Fellows et al., 2018).

Ubiquitination is involved in transcription initiation, elongation, silencing and DNA repair (Weake and Workman, 2008). H2A and H2B are the two most abundant ubiquitinated proteins in the nucleus, although others – such as H3, H4 and linker histone H1 – have also been reported to be modified by ubiquitin (Cao and Yan, 2012). H2A and H2B ubiquitination is implicated in transcriptional regulation. H2A ubiquitination is correlated with gene silencing, whereas H2B is mostly associated with transcription activation. Several studies suggest that DNA damage induces histone ubiquitination. It has been shown that ubiquitination of H2A by BRCA1/BARD1 promote homologous recombination (Densham et al., 2016), and ubiquitination by RNF168 seems to promote non-homologous end joining (Schwertman et al., 2016).

**Non-coding RNAs**

Non-coding RNAs (ncRNAs) represent a diverse family of regulatory transcripts that are transcribed from DNA, but not translated into proteins (Ounzain and Pedrazzini, 2015). ncRNAs can be classified based on their length as microRNAs (miRNAs), which are ∼22 nucleotides (nt) long, and long non-coding RNAs (lncRNAs), which are >200 nt long (Seeger et al., 2013). Several studies have demonstrated that miRNAs regulate a wide range of biological processes, including cell development, differentiation, proliferation, metabolism and apoptosis (Baltimore et al., 2008; Hwang and Mendell, 2006; Rottiers and Näär, 2012). Whereas lncRNAs display tissue-specific gene expression patterns, miRNAs are generally considered as an evolutionarily conserved group of ubiquitously expressed single-stranded RNAs that

act as negative post-transcriptional regulators via RNA silencing or translational inhibition. A significant number of studies have revealed the putative role of both classes of ncRNAs in conditions such as heart failure, cardiac autophagy, hypertension and various kidney disorders (Harvey et al., 2008; Lorenzen and Thum, 2016; Wing et al., 2013; Zhdanova et al., 2011). Many factors can alter the expression of ncRNAs, including point mutations, chromosomal amplification or deletion, modifications in promoter regions and activation of transcription factors.

**Table 1.**
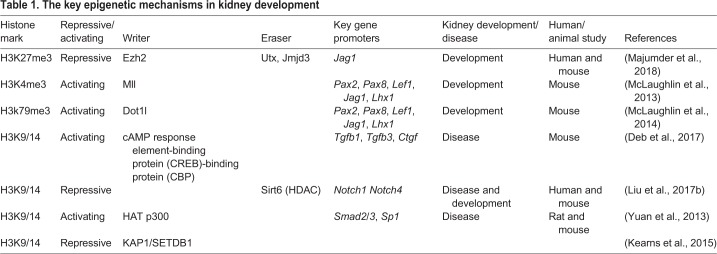
**The key epigenetic mechanisms in kidney development**

Defining the epigenetic landscape of renal progenitor cells is a difficult task. During embryonic kidney development, chromatin structure dynamically directs cell fate decisions by controlling the activation or repression of genes in a precise spatiotemporal manner (Dressler, 2009). Aberrant changes in the renal epigenetic signature could lead to congenital abnormalities of the kidney and urinary tract (CAKUT; Box 1), renal disease, and progression towards chronic kidney disease (CKD; Box 1) and end-stage renal disease (ESRD; Box 1). Importantly, CAKUT affect 1 in 500 infants and account for ∼25% of birth defects (Nicolaou et al., 2015). A high percentage of CAKUT cases are non-syndromic and exhibit phenotypic variability, leading to the speculation that these might have an epigenetic basis (Jin et al., 2014; Chen and El-Dahr, 2013; Hilliard and El-Dahr, 2016). Recent studies have shown differences in the epigenetic signatures between healthy and diseased populations (Beckerman et al., 2014; Chu et al., 2017; Ko et al., 2013; Reddy and Natarajan, 2015; Wing et al., 2014); however, whether these changes are the cause or consequence of the pathological state remains largely unknown. Thus, understanding the epigenetic landscapes of kidney development and disease could help to identify the key epigenetic changes happening under congenital and adult renal pathological states. These epigenetic hallmarks may serve as biomarkers of disease diagnosis and progression, as well as facilitate the development of novel therapeutic approaches targeting the epigenome.

Here, we summarize the most recent literature describing how epigenetic processes affect kidney development and disease, and discuss the interplay of these epigenetic events during renal dysfunction. We next introduce the potential use of kidney organoids derived from human pluripotent stem cells (hPSCs) combined with genome engineering technologies, particularly the clustered regularly interspaced short palindromic repeats (CRISPR)/CRISPR-associated protein 9 (CRISPR/Cas9; Box 1), as a novel approach for the targeted modification of the renal epigenome to study renal organ dysfunction, such as CKD.

## Epigenetic mechanisms in kidney development

The adult kidney is composed of many specialized epithelial, endothelial and stromal cell types; however, the functional components of the kidney, the renal epithelial cells, share a common lineage, the intermediate mesoderm (IM; Box 1), which is specified quite early in development. The IM arises soon after gastrulation and receives its name due to its specific location along the mediolateral axis of the embryo, as it lies between the axial (or somitic) mesoderm and the lateral plate mesoderm. During embryonic kidney development, two separate progenitor populations derive from the IM: the ureteric bud (UB; Box 1) and the metanephric mesenchyme (MM; Box 1) (Little et al., 2007). A subset of cells of the MM condenses around each UB tip to form the cap mesenchyme (CM; Box 1). Importantly, the CM forms the multipotent population of progenitors that will develop into the nephrons (Box 1), whereas the UB will constitute the collecting duct system (Box 1). All mature nephron cell types derive from a population of Six2^+^ renal progenitor cells that reside in the CM (Kobayashi et al., 2008).

Research on kidney development started with the pioneering work of Clifford Grobstein in 1956 (Grobstein, 1956). This early work showed that nephron formation requires a primary induction event from the UB to the CM. This inductive signal triggers mesenchymal-to-epithelial transition (MET; Box 1) within the CM, generating the renal vesicle, the precursor of the nephron. Canonical Wnt signaling (Box 1) is the major driving pathway that initiates nephrogenesis. Following Grobstein's work, the kidney has been widely investigated in various animal models, including mammals, frogs, fish and chicks (Dressler, 2006).

Globally, methylation of histones on lysine (K) or arginine (R) residues (Box 2) is a key epigenetic mechanism regulating gene expression during development and disease. Many of the promoters of lineage-specific genes in human and mouse embryonic stem cells (ESCs) are marked in a dual (bivalent) manner. In pluripotent stem cells (PSCs), the promoters of these genes are marked by H3 lysine 4 (H3K4me3; activating) and H3 lysine 27 (H3K27me3; repressive) marks (Table 1). This bivalent epigenetic status is believed to keep genes ‘poised’ for expression. Poised genes are either activated or repressed, depending on the appropriate differentiation trigger during development (Bernstein et al., 2006).

**Fig. 1.**
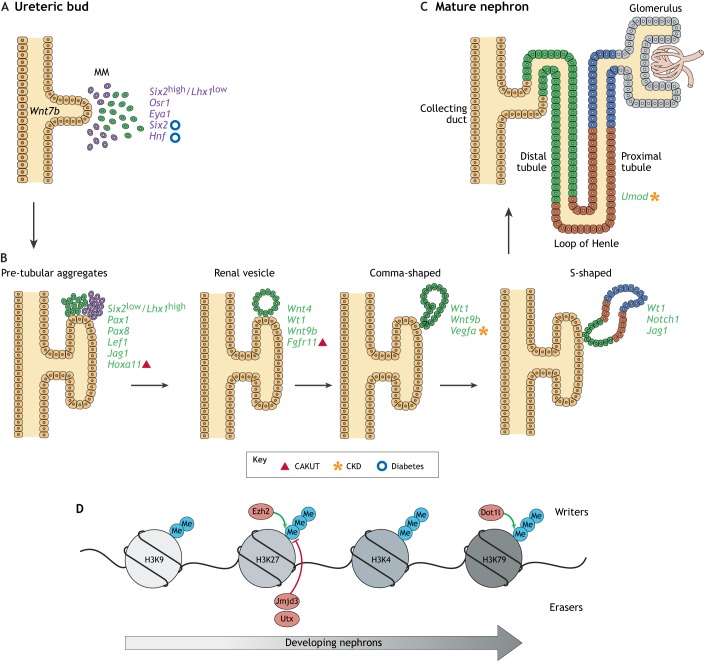
**Current knowledge of epigenetic modifications during renal development.** The definitive kidney arises from the interaction of two progenitor cell populations that evolve from the intermediate mesoderm. (A) The ureteric bud (UB) and the metanephric mesenchyme (MM) derive from the intermediate mesoderm early in development. Later, clusters of the MM condense to form caps around each UB tip. The quiescent *Six2*^high^/*Lhx1*^low^ cells are in purple. The activated *Six2*^low^/*Lhx1*^high^ cells (in green) condense around the tip, forming the cap mesenchyme (CM). (B) The different stages of nephrogenic body development: pre-tubular aggregate, renal vesicle, comma-shaped body and the S-shaped body. The markers specific to each stage are in green. The differentiation of pre-tubular aggregates into renal vesicles occurs through the process of mesenchymal-to-epithelial transition (Box 1). (C) Eventually, the distal end of the S-shaped body fuses with the UB (which forms the collecting duct) and the proximal end to form the vascularized glomerulus. (D) Histone marks distinctively dictate early kidney development and nephron differentiation and affect the expression of several developmental genes, several of which are involved in kidney disease. Methyl groups are indicated as ‘Me’. Adapted from Hilliard and El-Dahr (2016). This image is not published under the terms of the CC-BY license of this article. For permission to reuse, please see Hilliard and El-Dahr (2016).

Studies have shown that the differentiation of MM cells to nephron progenitors largely depends on two regulators, Six2 and the Wnt pathway (Park et al., 2012; Brown et al., 2013; Park et al., 2007). Although the epigenetic landscape of renal progenitor cells is not completely elucidated and more studies are needed, McLaughlin and colleagues started to tackle this matter in two studies. In the first study, the authors performed chromatin immunoprecipitation (ChIP)-DNA sequencing (ChIP-Seq; Box 1) with antibodies against H3K4me3 and H3K27me3 in immortalized MM mouse clonal cell lines representing the uninduced (self-renewing Six2^high^/Wnt^low^ – the mK3 line) and induced (Six2^low^/Wnt^high^ – the mK4 line) MM cell populations. The mK3 line expresses genes characteristic of the early mesenchyme, including *Hoxa11*, *Hoxd11*, *Twist1*, *Vim* and the Col1a gene family. Conversely, mK4 cells express genes characteristic of MET, such as *Pax8* or *Lhx1* (McLaughlin et al., 2013). The mK4 MM cell line showed a loss of the H3K9me2 and H3K27me3 repressive histone marks and retained the H3K4me3 activating mark on the promoters of nephrogenic lineage genes, such as *Pax2*, *Pax8*, *Lef1*, *Jag1* or *Lhx1* (Table 1; Fig. 1). In contrast, the activating H3K4me3 mark was depleted in proximal promoters of genes, such as *Six2*, *Osr1* or *Eya1*, in the mK3 uninduced MM cell line. Moreover, the authors searched the literature for candidate genes linked to CAKUT and glomerular filtration rate (GFR; Box 1), seeking the chromatin signature of these genes in the transition from the silent (mK3) to the transcriptionally active state (mK4). *Hoxa11* and *Fgfrl1* showed high H3K4me3 occupancy in both cell lines; *Blk* and *Wnt7b* presented loss of the repressive mark H3K27me3 and gain of the activating mark H3K4me3; *Hoxa13* and *Tsc* (also known as *Slc12a3*) lost the H3K4me3 active mark, whereas *Lrp2* and *Setdb1* genes gained the active mark H3K4me3 (McLaughlin et al., 2013). This study provides evidence about the importance of understanding the epigenetic mechanisms guiding nephrogenesis in order to further translate these findings in the field of CAKUT and other congenital kidney diseases. The second study by this group examined the spatial pattern of histone methylation and the developmental expression of the methyltransferases Ash2l and Ezh2/Suz12 (which add methyl groups to H3K4 and H3K27, respectively) and G9a (which methylates H3K9; also known as EHMT2). They used immunolocalization and real-time quantitative PCR in mouse kidneys at embryonic day 15.5. The CM showed a significant enrichment of H3K4me3, H3K9me3 and H3K27me3 in the *Six2* gene, indicating that this gene was poised for activation. In contrast, nascent nephron cells showed high levels of H3K4me3, and low levels of both H3K9me3 and H3K27me3, in the *Lhx1* gene. In addition, the authors showed that the generation of mature nephrons is characterized by an increase in H3K79me2/3 marks and by the upregulation of histone H3K79 methyltransferase Dot1l (McLaughlin et al., 2014) (Fig. 1).

In general, temporal changes in histone modifications are not restricted to embryogenesis. To investigate whether these epigenetic changes also occur in early postnatal life in mice, Lui and colleagues performed ChIP promoter tiling array (Box 1), comparing 1- to 4-week-old mice, to study temporal changes in the H3K4me3 and H3K27me3 histone marks at promoter regions throughout the kidney and lung genome. The authors found an association between temporal changes in histone methylation marks and changes in genes expression during juvenile life. Specifically, they found decreasing levels of H3K4me3 at promoters of cell cycle genes. Moreover, they found similarities in histone methylation in the kidney and lung, which prompted the authors to speculate about a common developmental program of histone methylation in multiple organs (Lui et al., 2014).

Remarkably, alterations in H3K27me3 levels in podocytes alter the Notch pathway (Box 1). This is due to the enrichment of H3K27me3 at the promoter region of the Notch ligand jagged 1 in these cells. Conversely, the de-repression of *Jag1*, which encodes jagged 1, facilitated podocyte dedifferentiation, increasing the susceptibility to focal segmental glomerulosclerosis (FSGS; Box 1). The same trend was observed in a mouse model of diabetic nephropathy (DN; Box 1) (Majumder et al., 2018). Notably, in the same study, inhibition of the demethylating enzymes JMJD3 and UTX (Table 1), and the resulting increase in H3K27me3, correlated with an attenuation of kidney injury in these models. More importantly, kidney tissue from patients with FSGS and DN showed reduced H3K27me3 levels, heightened UTX expression and increased levels of jagged 1. Indeed, the same group previously demonstrated that inhibition of the H3K27 trimethylating enzyme EZH2 causes podocyte injury in diabetic rats, suggesting that the H3K27me3 mark acts as a gatekeeper of podocyte function during development and disease (Siddiqi et al., 2016).

The paired domain transcription factor Pax2 is pivotal in the epigenetic specification of the definitive kidney and determines cell fate in the post-gastrulation embryo. In the developing mouse kidney, Pax2 specifies the IM that will generate the urogenital tract (Bouchard et al., 2002; Torres et al., 1995) and is expressed in the nephron progenitors. In human embryonic kidney (HEK293) cells, the modular BRCT-domain protein Pax transcription activation domain interacting protein (PTIP) and the mixed-lineage leukemia (MLL) complex (PTIP/MLL) links the Pax2 DNA-binding protein to the H3K4 methylation machinery (Patel et al., 2007). During kidney development, DNA-binding factors such as Pax2/8 could provide the locus and tissue specificity for histone methylation and chromatin modification, and thus determine the kidney-specific fate (Dressler, 2008). Another essential factor in kidney development and function is the transcription factor Wilms' tumor 1 protein (WT1). During development, WT1 responds to early Wnt9b inductive signals secreted by the UB at the renal vesicle stage (Fig. 1) (Carroll et al., 2005). Importantly, mutations in *WT1* lead to Wilms' tumor disease, the most common pediatric kidney cancer (Aiden et al., 2010; Rivera and Haber, 2005; Schedl and Hastie, 1998), which is characterized by diffuse Wilms' tumor precursor lesions exhibiting large active chromatin domains resembling those found in human ESCs (hESCs), together with a disturbed MET process (Aiden et al., 2010). Around 10% of sporadic Wilms' tumor specimens have inactivating mutations in *WT1*, and these same tumors often also contain mutations in β-catenin (*CTNNB1*) (Maiti et al., 2000). Indeed, mutant WT1 clones lack WNT/β-catenin signaling activity and fail to undergo renal differentiation (Royer-Pokora et al., 2010). When interrogating Wilms' tumor lesions, kidney tissue, and hESCs for genome-wide H3K4me3 and H3K27me3 occupancy, Aiden and colleagues showed that cells from Wilms' tumor lesions possess transcriptional programs resembling those found during early kidney development (Aiden et al., 2010). Recently, Essafi and colleagues found that WT1 activates the expression of Wnt4 by the recruitment of Cbp (also known as Crebbp) and p300 (also known as Ep300) transcriptional coactivators to the *Wnt4* locus. The switch in Wnt4 expression promoted by this mechanism, referred by the authors as ‘chromatin flip-flop’, is essential for the initiation of MET in nephrogenesis and may partially explain the ‘immature’ signature of Wilms’ tumor lesions (Essafi et al., 2011). More recently, other work has shown that DNA methyltransferase 3A (DNMT3A; Box 2) is a direct transcriptional target of WT1, suggesting that DNA methylation may be partly regulated by WT1 in Wilms' tumor cells (Szemes et al., 2013). Interestingly, the same work also proved that cellular WT1 levels can influence genome-wide DNA methylation of gene promoters. Overall, these findings suggest that Wilms' tumor cells have arrested development, and highlight potential therapeutic interventions that target epigenetic modifiers, such as histone methyltransferases (HMTs) and/or DNA methyltransferases.

Importantly, emerging evidence reveals that shifts in cellular metabolism affect the enzymes that control genome-wide epigenetic configuration, thereby modulating chromatin reorganization and gene expression changes during phenotypic reprograming and differentiation. Glycolysis (Box 1) generally accommodates a high rate of biosynthesis and cell proliferation, whereas oxidative phosphorylation (OXPHOS; Box 1) efficiently generates ATP to sustain the function of differentiated cells. Recent research showed the connection between metabolism and epigenetics in HEK293 cells. Specifically, overexpression of the pluripotency marker Lin28A (Box 1) in this cell line reprogrammed the cellular energetics through hexokinase II and, consequently, increases in glycolysis. These changes maintained a dedifferentiated, stem-like phenotype in these cells (Docherty et al., 2016).

Little is known about the function of non-coding RNAs, such as microRNAs (miRNAs) (Box 1) and lncRNAs, in human kidney development. To date, seminal studies have identified the significance of miRNA-mediated gene expression in renal development and disease in mouse models. Blocking miRNA synthesis in murine renal cell lines suggested their role as epigenetic regulators (Bartram et al., 2013; Ho et al., 2012; Nagalakshmi et al., 2011; Patel et al., 2007; Sequeira-Lopez et al., 2010). Importantly, studies in conditional *Dicer1* knockout mice, whereby the miRNA biogenesis enzyme Dicer1 (Box 1) was removed from the UB epithelial compartment, confirmed the key role of global miRNA-mediated gene regulation in the UB (Bartram et al., 2013; Nagalakshmi et al., 2014; Nagalakshmi et al., 2011; Pastorelli et al., 2009; Patel et al., 2007). Similarly, conditional deletion of *Dicer1* in mature podocytes resulted in glomerular abnormalities, such as glomerulosclerosis and fibrosis (Harvey et al., 2008; Zhdanova et al., 2011), and podocyte-specific deletion of *Drosha* (Box 1) resulted in collapsing glomerulopathy comparable to the phenotype of *Dicer1* knockouts (Zhdanova et al., 2011). Furthermore, the miRNA synthesis pathway seems to be important for the survival of the nephron progenitor population during development. Nagalakshimi and colleagues elegantly showed this *in vivo* by ablating *Dicer1* function specifically from nephron lineage cells (Six2-Cre-mediated) and from cells of the UB-derived collecting duct system (HoxB7-Cre-mediated). The authors demonstrated that Six2-Cre-mediated removal of *Dicer1* resulted in elevated apoptosis and premature termination of nephrogenesis, and thus confirmed that *Dicer1* is important for maintaining the viability of the Six2 self-renewing progenitor pool and, consequently, for the development of a normal nephron complement. Conversely, HoxB7-Cre-mediated removal of *Dicer1* caused the development of renal cysts in this model (Nagalakshmi et al., 2011) Independent studies have also shown the effects of *Dicer1* deletion in Foxd1^+^ progenitor cells (Box 1), which give rise to renal stroma (Nakagawa et al., 2015; Phua et al., 2015). *Dicer1* inactivation in the Foxd1^+^ cortical stroma results in multiple defects of nephrogenesis (Nakagawa et al., 2015; Phua et al., 2015), including expansion of nephron progenitors, a decrease in renin-expressing cells, fewer smooth muscle afferent arterioles, and progressive mesangial cell loss in mature glomeruli (Phua et al., 2015).

Despite the knowledge acquired from mouse models of miRNA regulation in kidney development, the specific roles of individual miRNAs remain largely unknown. However, Marrone and colleagues identified two miRNAs (miR-17/92) associated with Feingold syndrome, a developmental defect in humans. The authors generated a transgenic mouse with a conditional deletion of the miR-17/92 cluster in the nephron progenitor pool and their derivatives (Six2 cells and their Six2-derivatives, respectively). Although the nephron progenitor population was preserved in these mice, miR-17/92 deletion impaired proliferation and reduced the number of developing nephrons. Interestingly, mutant mice developed signs of renal disease. Overall, this study supports a role for the miR-17/92 cluster in the regulation of nephron development, with later consequences for renal function in adult mice (Marrone et al., 2014).

## Epigenetics of kidney disease

In 2012, the Kidney Disease Improving Global Outcomes (KDIGO) consortium defined CKD as abnormal kidney structure or function lasting for at least 3 months and having implications for health (International Society of Nephrology, 2013). The final stage of CKD is ESRD, at which point the patient needs a renal transplant to stay alive. Currently, there are very few therapeutic options for people suffering from CKD. In addition, screening measures that can identify patients with increased risk of renal disease are currently lacking. Over the past decade, genome-wide association studies (GWAS; Box 1) have improved our understanding of CKD. Köttgen and colleagues performed the first meta-analysis in CKD by analyzing more than 200,000 individuals, 2,400 of whom had CKD. The genes that related to renal phenotypes were *UMOD*, *SHROOM3* and *STC1* (Köttgen et al., 2010). After this first study, Chambers and colleagues demonstrated the association between the loci reported by Köttgen together with markers of kidney function. Interestingly, the relationships between these parameters were similar amongst people with and without diabetes or hypertension (Chambers et al., 2010). Additional loci identified in GWAS analysis for renal function and CKD included *SLC34A1*, *VEGFA* or *SLC7A9*, among others (Chambers et al., 2010; Pattaro et al., 2012). We refer the reader to several reviews for details (Böger and Heid, 2011; Köttgen et al., 2018; Piras et al., 2017; Smyth et al., 2014). DNA sequence variants in many genes, as well as environmental factors and their interactions, influence CKD susceptibility (Chu et al., 2017). So far, GWAS have successfully identified more than 60 genetic loci that associate with kidney function and CKD (Köttgen et al., 2010; Okada et al., 2012; Pattaro et al., 2012, 2016). Understanding how these heritable components predispose to CKD and its progression towards ESRD remains a major challenge in the field. However, many aspects are still unclear, and understanding the epigenetic modifications associated with CKD may pave the way towards innovative approaches targeting renal dysfunction. Many factors can affect the epigenome in CKD, but here we comment on those related to aging, diabetes, acute kidney injury (AKI) and hypoxia (Box 1).

### Aging

Many clinical studies have shown that changes in DNA methylation are implicit in the development of CKD (Chu et al., 2017; Ko et al., 2013; Pushpakumar et al., 2015; Wing et al., 2014). However, understanding how these changes relate to aging needs further investigation. To date, CKD has been strongly associated with age, with up to 30% of people aged 70 years and older affected by CKD (Weinstein and Anderson, 2010). Aging is a complex phenomenon associated with physiological alterations in the function of cells and organs over time. At the epigenetic level, aging is associated with an altered epigenomic state called epigenetic drift, which reflects a deficient maintenance of epigenetic marks that leads to impaired cellular function in aged cells. Other features of aging are the upregulation of proinflammatory cytokines, telomere shortening, accumulation of senescent cells and impaired mitochondrial metabolism (Michaud et al., 2013; Shammas, 2011; Sun et al., 2016; van Deursen, 2014). Each one of these features is regulated, in part, by epigenetic mechanisms and could, in the context of the kidney, be associated with CKD. For instance, cyclin-dependent kinase inhibitor 2A (CDKN2A; also known as p16), a validated biomarker of aging that acts as an off switch for cell proliferation, has been identified as a predictive marker of renal function in kidney biopsies (Gingell-Littlejohn et al., 2013). Interestingly, CDKN2A expression is regulated by the antisense lncRNA antisense non-coding RNA in the INK4 locus (ANRIL; also known as CDKN2B-AS1), which interacts with the H3K27 methylation machinery to repress *CDKN2A* expression (Yap et al., 2010). Telomere attrition is associated with aging in CKD (Carrero et al., 2008), and the lncRNAs telomerase RNA component (TERC) and telomeric repeat-containing RNA (TERRA) regulate telomere length during cellular aging (Montero et al., 2018). Although a recent review (Shiels et al., 2017) describes many of these associations in detail, there is a lack of studies that address a direct relationship between epigenetic regulation, aging and renal disease. Thus, gaining further insight into how this process affects renal biology is critical, with the final aim of designing therapies targeting epigenetic modifications that have arisen during CKD, which are also common to the aging process.

It has long been established that loss of DNA methylation (Box 2) and associated heterochromatin is a major feature of aging. One of the genes that link epigenetics, aging and their association with CKD is *KL*, which encodes the transmembrane protein klotho. Klotho serves as the cofactor for fibroblast growth factor 23 (FGF-23) to bind to its cognate receptor and regulate phosphorus and vitamin D metabolism. The soluble form of klotho has anti-aging properties, which may be mediated via multiple systemic effects, including regulation of insulin signaling (Rubinek et al., 2016; Silva et al., 2017), oxidative stress (Lim et al., 2017; Liu et al., 2017a), and fibrosis (Doi and Masaki, 2017). Decreased *Kl* expression, lower klotho levels in kidney tissue, and lower circulating levels of soluble klotho have been identified in animal models of CKD, suggesting the implication of klotho deficiency in CKD (Chen et al., 2013; Gołembiewska et al., 2016) and in aging-related disorders when its promoter is aberrantly methylated (Kuro-O et al., 1997). Recent studies further suggest that *KL* promoter hypermethylation is associated with the pathogenesis of acute kidney disease and CKD. These studies provide a molecular basis for the epigenetic intervention targeted to the *KL* promoter for the treatment of kidney diseases (Doi and Masaki, 2017; Zhang et al., 2016, 2017).

### Diabetes

Approximately 20–30% of patients with diabetes will eventually develop DN, which is characterized by different pathological changes compromising renal function, such as renal glomerular hypertrophy, expansion of mesangial and tubular compartments, and podocytopenia. DN is clinically identified by albuminuria, rising creatinine levels and abnormal glomerular filtration rates. The molecular mechanisms by which some patients develop DN are still not fully understood. Many clinical studies have shown that strict glycemic control early on in patients newly diagnosed with diabetes can reduce the incidence of DN at a later stage of the underlying disease. The Diabetes Control and Complications Trial was the first to define this phenomenon as metabolic memory (Diabetes Control and Complications Trial Research Group et al., 1993). Metabolic memory is induced by irreversible changes in oxidative stress, non-enzymatic glycation of proteins, epigenetics and chronic inflammation (Testa et al., 2017; Tonna et al., 2010). However, the molecular mechanisms that can explain these changes remain unknown.

Emerging evidence suggests the critical role of epigenetic mechanisms in the pathogenesis of DN. The impact of DNA methylation on DN has raised attention in the past 10 years. A study by Ko and colleagues analyzed DNA methylation in kidney tubular epithelial cells, and showed significant differences in the methylation of core pro-fibrotic gene enhancers, correlating with downstream transcript levels (Ko et al., 2013). Interestingly, the authors found that differentially methylated regions contain consensus binding motifs recognized by key kidney developmental factors, including SIX2, HNF and TCFAP (also known as TFAP) proteins (Ko et al., 2013). Of note, the CKD patient group in this study included both hypertensive and diabetic CKD patients, whereas the control group included diabetic patients. Overall, Ko and colleagues observed that 70% of the differentially methylated regions were hypomethylated in CKD; however, such findings could be related to the experimental data set. Similarly, a comparative study interrogating the DNA methylation status from the saliva of type 2 diabetes patients with or without ESRD identified 187 genes for which mean methylation levels differed between the two groups, and that were also involved in kidney development (Sapienza et al., 2011). The associations between DNA methylation, CKD and kidney development could provide the mechanistic link between fetal reprograming and CKD. In the field of epigenetics in kidney disease, few studies indicate that epigenetic differences might play a role in CKD development. Animal model studies indicate that calorie, protein or oxygen restriction are associated with lower nephron number, hypertension and microalbuminuria (Hoppe et al., 2007). These findings have led some authors to speculate that the epigenetic differences established during development might mimic the changes observed in diabetic patients with kidney damage (Ko et al., 2013).

Apart from DNA methylation, recent studies have implicated histone deacetylases (HDACs; Box 2) in diabetes and associated microvascular diseases. Deb and colleagues showed that exposure of murine glomerular mesangial cells to high glucose-induced cAMP response element binding protein (CREB)-binding protein (CBP)-mediated H3K9/14 hyperacetylation in ∼5000 gene promoters, including those of the major pro-fibrotic factors (Table 1) (Deb et al., 2017). The sirtuin (SIRT) family (Table 1) also plays an important role in histone deacetylation in kidney disease, and several studies suggest SIRT as a potential therapy target in DN. Liu and colleagues showed that advanced glycation end products (AGEs; Box 1) induced p65 (also known as RELA) and STAT3 acetylation in human podocytes, causing proteinuria and kidney injury due to decreased activity of SIRT1 (Liu et al., 2014). Recently, others have demonstrated that SIRT6, a well-studied H3K9 deacetylase (Table 1; Box 2), was downregulated in renal biopsies from DN patients compared with subjects without diabetes or renal disease. Accordingly, when the authors measured the levels of H3K9 acetylation, they found a significant increase in H3K9ac levels in renal biopsies from patients with DN (Liu et al., 2017b).

As discussed above, miRNAs have a role in nephrogenesis, but most of the studies published until now have been focused on pathologies such as renal carcinoma, DN, cystogenesis and glomerulopathies (Petrillo et al., 2017). In DN, some miRNAs have been identified as biomarkers of disease gestation and progression (Al Kafaji and Al Muhtaresh, 2018; Eissa et al., 2016; El-Samahy et al., 2018; Ghai et al., 2018; Li et al., 2018; Park et al., 2018; Yu et al., 2018). The identification of metabolic memory in DN suggests the need for very early intervention to target the microvascular complications that arise during diabetes. For this reason, having reliable biomarkers for early detection of DN has an important value in the clinic. In this regard, Eissa and colleagues screened miRNA expression in urinary exosomes from a homogenous cohort of type 2 diabetes patients classified according to the presence of normo-, micro- and macroalbuminuria. In this study, miR-133b, miR-342 and miR-30a were highly expressed in type 2 diabetes patients compared with healthy controls. Moreover, their expression correlated with hemoglobin A1c (HbA1c; Box 1), systolic-diastolic blood pressure, low-density lipoprotein, serum creatinine, urinary albumin creatinine ratio and eGFR. The most important aspect of this study was that the three miRNAs were altered in all experimental groups, indicating their usefulness as early molecular indicators before the onset of albuminuria (Eissa et al., 2016). Apart from being potential biomarkers for DN, there is increasing evidence demonstrating that miRNAs act as key players in DN pathology (Bai et al., 2018; Deshpande et al., 2018; Guo et al., 2017b; Sun et al., 2018; Zhang et al., 2018).

### Hypoxia

The kidney is the second organ after the heart in terms of number of mitochondria. Any damage to the kidney will be reflected in the mitochondrial function in kidney cells; specifically, in the renal tubular epithelial cells, which are among the most energy-demanding cells in the human body. Owing to their high level of specialization in aerobic metabolism, they are particularly sensitive to oxygen alterations, to which they respond by decreasing OXPHOS, resulting in an increased production of mitochondrial reactive oxygen species (ROS). Hypoxia is one of the main hallmarks of the beginning of CKD. Moreover, hypoxia also induces epigenetic changes. The oxygen molecule is required for the activity of the ten-eleven translocation (TET) enzymes, oxidation enzymes that catalyze the conversion of the modified DNA base 5-methylcytosine (5-mC) to 5-hydroxymethylcytosine (5-hmC) (Tahiliani et al., 2009). Bechtel and colleagues found that DNA methylation in kidney fibroblasts determines fibrogenesis, specifically via hypermethylation of the *RASAL1* promoter (Bechtel et al., 2010). Subsequently, Tampe and colleagues demonstrated that the induction of endogenous Tet3/Tdg-mediated DNA demethylation activity by dihydralazine treatment reversed aberrant hypermethylation of *RASAL1* (Tampe et al., 2015). Although there are no studies that directly link DNA methylation status with hypoxia in CKD, work in kidney cancer has shown that hypoxia-induced loss of TET activity increases hypermethylation at gene promoters *in vitro* (Thienpont et al., 2016). These findings suggest that hypoxia could be one of the factors that modify DNA methylation profile in CKD. Under hypoxia, kidney cells can adapt to changes in oxygen tension in order to survive by activating hypoxia-inducible factors (HIFs), among others. In 2012, Mimura and colleagues performed ChIP-Seq to determine the genome-wide binding sites of HIF-1 (also known as HIF1A) in human umbilical vein endothelial cells (HUVECs) under hypoxia. The authors demonstrated that HIF-1 and lysine-specific demethylase 3A (KDM3A; Box 2) are recruited to the glucose transporter 3 (*GLUT3*; also known as *SLC2A3*) gene locus to cooperatively demethylate H3K9me2 and construct long-range looping interactions between the promoter and the distal enhancer, maximizing the hypoxic induction of GLUT3 and, consequently, increasing glucose uptake (Mimura et al., 2012). Also, the same group identified that the upregulation of aspartyl-tRNA synthetase anti-sense 1 (DARS-AS1), a novel lncRNA, was HIF-1 dependent and inhibited apoptosis in renal tubular epithelial cells cultured under hypoxia (Mimura et al., 2017). Another mechanism of hypoxia-induced epigenetic changes is through impairing the expression and activity of Dicer1 (Ho et al., 2012). In breast cancer cells, DICER1 expression is suppressed through the inhibition of the oxygen-dependent H3K27me3 demethylases KDM6A/B, which silence the *DICER1* promoter (van den Beucken et al., 2014). In a human proximal tubular epithelial cell line (HK2), Du and colleagues performed array-based miRNA profiling under hypoxia and normoxia for 24 h and 48 h. Among a panel of seven miRNAs that were downregulated in hypoxia, the authors identified miR-34a as the most downregulated. The functional role of miR-34a was assessed by transfections of miR-34a inhibitors, which promoted the expression of mesenchymal markers, such as alpha smooth muscle actin and vimentin. Because miR-34a targets include NOTCH1 and JAG1, the authors demonstrated that downregulation of JAG1 or NOTCH1 using exogenous small interfering RNAs (siRNAs) effectively prevented miR-34a inhibitor-induced epithelial-to-mesenchymal transition. These results provide evidence that hypoxia-induced decrease of miR-34a expression could promote epithelial-to-mesenchymal transition in renal tubular epithelial cells by de-repressing NOTCH1 and JAG1 (Du et al., 2012).

Interestingly, when pVHL, the protein product of the von Hippel-Lindau (*VHL*) tumor suppressor gene, is inactivated, HIFs are constitutively active and drive tumorigenesis and growth. Alterations in *VHL* are the most prevalent molecular features of clear cell renal cell carcinoma (ccRCC; Box 1), a major subtype of kidney cancer. *VHL* inactivation occurs in 80–90% of ccRCC cases, and other tumor suppressors that are recurrently inactivated in CCRC are readers, writers and erasers of key histone marks on the tails of histones H2A and H3 (Table 1). Specifically, PBRM1 recognizes H3K14Ac, BAP1 deubiquitinates K119ub on H2A, SETD2 methylates H3K36Me3, JARID1C demethylates H3K4Me3 and UTX demethylates H3K27Me3 (Davidowitz et al., 2001; Gossage et al., 2015; van Houwelingen et al., 2005). Epigenetic alterations and transcriptional deregulation are thus central to ccRCC. Preclinical studies have shown that treatment with the HDAC inhibitor vorinostat augmented the activity of the mTOR inhibitor temsirolimus to induce apoptosis in xenografted ccRCC cell lines through suppression of survivin levels (Mahalingam et al., 2010). Despite these findings, a phase II clinical trial of a different HDAC inhibitor, panobinostat, in 20 patients with refractory ccRCC, failed to show an objective response (Hainsworth et al., 2011). These results indicate the need to develop new knowledge on the role of epigenetics in this type of kidney cancer.

### AKI

AKI is characterized by an abrupt decline in kidney function, which involves two main phases. The first phase after the initial insult includes cell death and inflammation. Then, in the second phase, the recovery of function and structure can take place or, when the injury is too severe, result in a transition to CKD (Basile et al., 2012). Although the molecular mechanisms underlying AKI remain unclear, epigenetic modifications driven by aging, diabetes and hypoxia have been suggested to contribute to the progression of AKI towards CKD (Guo et al., 2017a; Jain et al., 2017; Nangaku et al., 2017; Silveira Santos et al., 2018; Tampe et al., 2017; Thakar et al., 2011).

Several studies have recently suggested the involvement of DNA methylation changes in kidney diseases. For instance, Bechtel and colleagues challenged CD1 mice with folic acid to induce fibrosis, and then treated a subgroup with the demethylating agent 5′-azacytidine (Bechtel et al., 2010). The authors demonstrated that progressive fibrosis and kidney failure were significantly inhibited in mice that received 5′-azacytidine from day 3 to day 28 after folic acid challenge. These observations indicated the protective role of DNA demethylation in kidney fibrosis (Bechtel et al., 2010). On the contrary, Guo and colleagues examined the effects of the DNA methylation inhibitor 5-aza-2’-deoxycytidine and showed that it increased cisplatin-induced apoptosis in a rat kidney proximal tubular cell line. Furthermore, using a kidney proximal tubule (PT)-specific DNMT1 (PT-DNMT1) knockout mouse model, these authors showed a more severe AKI response to cisplatin treatment in the knockout compared with wild-type mice, suggesting that DNA methylation is protective in cisplatin-induced AKI (Guo et al., 2017a). These conflicting results highlight the possibly different roles of DNA methylation in AKI, fibrosis or CKD, and underscore the need to precisely define the role of DNA methylation in AKI. This knowledge would pave the way to the discovery of novel therapeutic interventions based in modifiers of DNA methylation.

Histone acetylation is a protective mechanism against cisplatin-induced nephrotoxicity (Arany et al., 2008; Dong et al., 2010; Ranganathan et al., 2016). Recently, Ruiz-Andres and colleagues found that histone crotonylation (Box 1), a robust indicator of active promoters (Tan et al., 2011), might be a contributor in AKI. The levels of histone crotonylation were increased in kidney tissue from animals treated with folic-acid, which induced AKI. The crotonylation levels were also increased in murine proximal tubular cells treated with the inflammatory agent TWEAK. Moreover, the authors identified *Pgc1a* (also known as *Ppargc1a*) and *Sirt3* as potential target genes of histone crotonylation (Ruiz-Andres et al. 2016).

Taken together, these studies show that epigenetic (de)regulation has important, although not yet completely understood, implications in kidney disease. The following sections discuss new promising approaches to improve our understanding of these implications.

## Kidney organoids from hPSCs

**Fig. 2.**
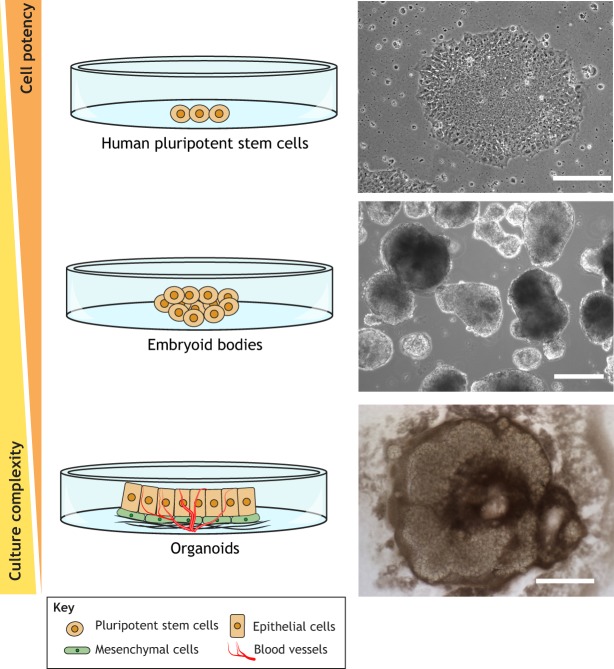
**hPSC differentiation approaches.** Undifferentiated hPSCs can be expanded under standard maintenance culture conditions (top), and subsequently differentiated using different methodologies (Morizane et al., 2015; Qian et al., 2016; Takasato et al., 2015). The formation of embryoid bodies (middle) recapitulates early developmental events, including the three germ layers – the ectoderm, mesoderm and endoderm. Recent advances in 3D culture techniques have allowed the generation of organoids (bottom), which possess high tissue complexity and tissue-specific functional activity. These include layers of differentiated cells in close cell­–cell and cell–extracellular matrix contact. One of the important challenges in the organoids field is the formation of a vasculature. To date, only two studies of kidney organoids have shown the expression of vasculogenesis markers (Takasato et al., 2015; Czerniecki et al., 2018). Scale bars: 200 µm (top and middle), 500 µm (bottom). Contrast-phase images are courtesy of E.G.

One of the major roadblocks in developing therapies for CKD has been the lack of reliable preclinical models. For many diseases, mice have been instrumental in understanding the mechanisms underlying pathogenesis, but their utility in studying CKD has been limited because they fail to recapitulate important functional, structural and molecular features of advanced human kidney disease. In this regard, hPSCs represent an unprecedented resource for the generation of functional renal cell types suitable for disease modeling and cell replacement therapies. Typically, differentiation of hPSCs starts with the induction of formation of embryoid bodies (EBs; Box 1; Fig. 2). Their three-dimensional (3D) shape and the establishment of complex cell–cell adhesions and paracrine signaling are responsible for their differentiation into specific cell types. Although EBs can recapitulate several aspects of early development (reviewed in Lancaster and Knoblich, 2014), there are still several limitations that hamper the translation of these differentiation approaches into a clinical setting (i.e. derivation of limited cell types, low reproducibility and scalability, and high variability between different cell lines, among others). Nevertheless, the knowledge obtained from EB differentiation approaches has provided instrumental cues for the proper instruction of hPSCs toward different cell types. More importantly, the EB technology has been fundamental for the guiding hPSCs to form 3D self-organized tissue-specific derivatives, or organoids, from different tissues, including the kidney (reviewed in Koehler et al., 2017; Lancaster and Knoblich, 2014; Qian et al., 2016) (Fig. 2).

In the past 5 years, several studies have provided experimental evidence that hPSC-derived organoids can recapitulate several biological processes related to spatial and temporal organization of multiple tissue-specific cell types within these 3D structures. Of note, organoids exhibit physiological functions close to their *in vivo* equivalents (Morizane et al., 2015; Qian et al., 2016; Takasato et al., 2015). However, hPSC-derived organoids transcriptomically match with embryonic-related tissues rather than their adult counterparts, which limits further applications aiming to model age-related disorders (Takasato et al., 2015), such as CKD. Most hPSC-derived organoids still fail to provide complex tissue interactions, such as innervation and vascularization, which prevents the proper control of cell–cell interactions, cell–matrix interplay and cell organization within the organoid. Nevertheless, the latest advances in the field highlight the use of these systems as powerful models for future clinical applications, including nephrotoxicity screening, disease modeling and cell replacement therapies in kidney disorders.

### Modeling kidney diseases in kidney organoids

To fulfil their potential for functional studies and serve as instrumental platforms for modeling kidney development and disease, organoid technology must rely on straightforward approaches that can overcome the major limitations accounted so far (i.e. embryonic transcriptional profile, lack of proper vasculature and innervation). Indeed, kidney organoids grown from patient-derived induced pluripotent stem cells (iPSCs) may not reflect disease-relevant phenotypes, but instead they represent the variation in the genetic backgrounds from the starting cell population (Soldner and Jaenisch, 2012). Genome editing approaches can efficiently introduce specific genetic alterations into the genome of hPSCs to generate isogenic starting stem cell lines. In this manner, gene-edited hPSC lines would only differ in disease-related gene mutations compared with the parental line. This strategy may also bypass problems with systemic variations that arise not only from the reprogramming process itself, but also from the hPSCs' differentiation potential into disease-relevant cell types. Although the scientific community is yet to undertake the use of kidney organoids for modeling secondary renal pathologies, such as DN, recent studies successfully recapitulated primary kidney disease phenotypes in kidney organoids generated from either patient-derived iPSCs or from genetically engineered human embryonic stem cells (hESCs). The first report recapitulating a primary kidney disease-related phenotype used iPSCs from patients affected by autosomal dominant polycystic kidney disease (ADPKD; Box 1). In this study, Freedman and colleagues showed that ADPKD iPSCs, as well as somatic epithelial cells and hepatoblasts/biliary precursors differentiated from these cells, expressed lower levels of polycistin-2 (PC2), encoded by the *PKD2* gene (Freedman et al., 2013). Later, the same group used kidney organoids from genetically engineered hESCs for the introduction of kidney-related mutations to ascertain kidney disease-related phenotypes in an isogenic background. The authors applied the then new CRISPR/Cas9 system to inactivate the expression of podocalyxin (*PODXL*), *PKD1* and *PKD2* by transiently transfecting undifferentiated hPSCs with plasmids expressing the Cas9 nuclease and the synthetic chimeric guide RNAs (gRNAs) targeting these genes. Upon clonal expansion of the biallelic knockout lines, they further differentiated these targeted hPSC clonal lines for functional analysis. *PODXL*-defective kidney organoids exhibited junctional organization defects in podocyte-like cells, while *PKD1* or *PKD2* knockout organoids showed cyst formation from kidney tubules (Freedman et al., 2015), a phenotype similar to ADPKD. More recently, a proof-of-concept study validated the use of kidney organoids for diseases modeling. Specifically, Forbes and colleagues performed whole-exome sequencing of a patient with nephronophthisis (NPHP) and their parents, identifying compound-heterozygous variants in *IFT140*, a gene previously associated with NPHP-related ciliopathies. Using reprogramming and CRISPR/Cas9 editing, the authors generated patient-derived and gene-corrected isogenic control iPSCs from skin fibroblasts of the individual affected by compound-heterozygous *IFT140* variants, and differentiated both iPSC lines into kidney organoids. Within the tubular epithelium of unedited *IFT140* mutant organoids, a classical ciliary morphology indicative of retrograde IFT dysfunction was identified. Gene-corrected organoids demonstrated a capacity to resolve this ciliary morphology, thereby verifying the genomic variant as disease causing. Additionally, transcriptional profiling and differential gene expression analysis, comparing mutant and gene-corrected organoids, revealed dysfunctional pathogenetic pathways not previously described in *IFT140*-deficient disease models but characteristic of other NPHP genes, suggesting that this model could clarify the common pathogenetic mechanisms for this heterogenetic rare disease (Forbes et al., 2018). One of the immediate applications of hPSC-derived kidney organoids has been nephrotoxicity screening (Morizane et al., 2015; Qian et al., 2016; Takasato et al., 2015). Czerniecki and colleagues have recently established a robotic pipeline to produce and analyze kidney organoids in a microwell format that helped the authors to improve differentiation outcomes, screen for toxicity and comprehend disease (Czerniecki et al., 2018).

### Modeling epigenetic modifications in kidney organoids

**Fig. 3.**
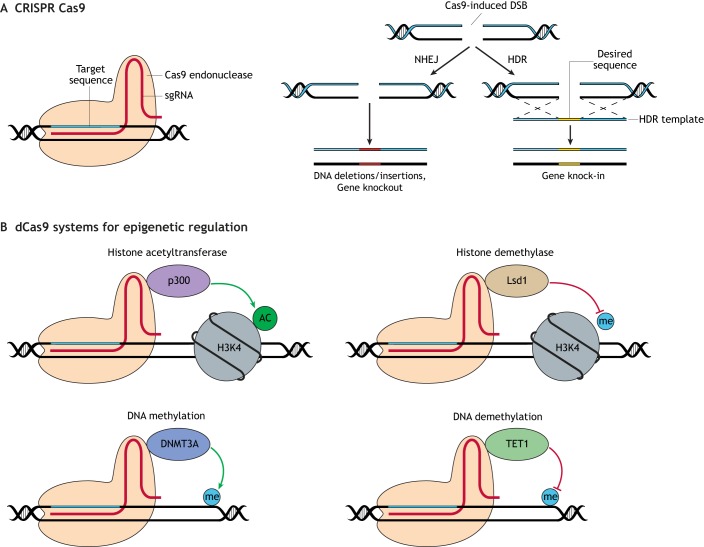
**CRISPR/Cas9-based approaches for modeling kidney disease.** (A) CRISPR/Cas9-based genome editing. CRISPR/Cas9 genome editing requires two components for sequence-specific DNA cleavage: a single-guide RNA (sgRNA) targeted to the genomic locus of interest and the RNA-guided Cas9 endonuclease. In the presence of an sgRNA, the Cas9 endonuclease homes in on the target sequence and induces a double-stranded break (DSB) directly upstream of a protospacer-adjacent-motif (PAM) sequence. The DSB is repaired by cell-endogenous mechanisms, either the non-homologous end joining (NHEJ) or homology-directed repair pathways (HDR). NHEJ does not require a donor template and creates insertion or deletion mutations that usually produce knockout phenotypes. The HDR pathway allows precise modifications of the genome in the presence of a donor template to generate knock-in phenotypes. (B) dCas9 systems for epigenetic editing. dCas9 can be fused to histone modifiers, such as the histone acetyltransferase p300, which specifically acetylates histones near the target DNA sequence, activating gene expression (Hilton et al., 2015), or the histone demethylase Lsd1 (Gao et al., 2014). Additionally, dCas9 fusion systems can also modulate DNA methylation levels with DNMT3A or TET1 (Liu et al., 2016). Acetyl groups are indicated as 'AC'; methyl groups are indicated as ‘me’.

In recent years, researchers generated organoid models derived from reporter PSCs lines using genome-engineering strategies. These engineered organoids facilitate the identification of lineage-specific cell types within the organoid and, more importantly, enable the tracking of the morphological changes during differentiation (Forster et al., 2014; González et al., 2014; Kadoshima et al., 2013; Nakano et al., 2012; Wataya et al., 2008; Zhu et al., 2016). With CRISPR/Cas9, researchers can now introduce a fluorescent reporter under the endogenous regulation of a cell-type-specific promoter, avoiding undesired responses due to the non-physiological genetic context. In this manner, the organoid technology would benefit from large-scale screens to test the optimal conditions for hPSC differentiation and maturation under different metabolic environments mimicking physiological and pathological conditions. Remarkably, it has already been shown that it is possible to target the *Cas9* gene into the mammalian safe harbor locus *AAVS1* (Box 1) to generate Cas9-expressing cells for further applications (Fig. 3), including the generation of multiple knockouts, introduction of single-nucleotide alterations, as well as inducible knockouts during hPSC differentiation (González et al., 2014). Specifically, the inducible CRISPR (iCRISPR; Fig. 3) system is based on the generation of inducible Cas9 (iCas9) hPSCs that express the endonuclease Cas9 upon doxycycline (Box 1) treatment. In this manner, upon transient transfection of single-guide RNAs (sgRNAs) to iCas9 hPSCs and doxycycline treatment to induce expression, Cas9 is directed for site-specific DNA cleavage, efficiently generating mutant hPSCs lines (González et al., 2014). This approach may also facilitate the generation of more complex genomic modifications, such as reporter alleles or knock-ins via homology-directed repair (HDR)-mediated gene targeting using long donor DNA templates encoding protein tags, fluorescent proteins or disease-specific variant sequences (González et al., 2014; Zhu et al., 2016).

The application of CRISPR/Cas9 technology (Box 1) to kidney organoids represents an unprecedented opportunity to target specific DNA sequences related to CKD and other kidney diseases. Of note, these technologies may soon allow researchers to study kidney-associated systemic disorders, such as diabetes and aging. The use of deactivated Cas9 (dCas9) fused to functional transcriptional repressors, like Krüppel-associated (KRAB) (Gilbert et al., 2013) or the tripartite activator VP64-p65-Rta (VPR) module (Chavez et al., 2015), may open new venues for the simultaneous activation or repression of endogenous coding and non-coding genes. These sequences can be activated or inactivated in a controlled manner, and in culture conditions that mimic disease, such as the diabetogenic environment or fibrotic insults. Additionally, Liu and colleagues have repurposed the CRISPR/Cas9 system to edit DNA methylation by fusing dCas9 with TET1 and DNMT3A (Box 2; Fig. 3), which are involved in DNA methylation/demethylation pathways, respectively (Liu et al., 2016). With this approach, it would be possible to identify and validate how changes in DNA methylation correlate with kidney function and CKD. The use of epigenome-wide association studies (EWAS) has identified genome-wide differences in DNA methylation at CpG islands with single-base resolution, discerning differentially methylated regions from the whole blood of patients with reduced eGFR, CKD and renal fibrosis (Chu et al., 2017). Researchers could use the aforementioned dCas9-TET1 or dCas9-DNMT3A systems to validate the functional implications of these differentially methylated regions to improve our mechanistic understanding of CKD.

The functional validation of single-nucleotide polymorphisms (SNPs) could also soon benefit from the organoid technology combined with CRISPR/Cas9 genome engineering. One possibility to identify target genes for distal candidate functional SNPs would be to modulate the chromatin state of the regulatory element with dCas9 fused to a chromatin-modifying domain (Fig. 3). For example, the histone demethylase KDM1A (LSD1) and a KRAB domain that recruits KAP1/SETDB1 histone methyltransferase (Table 1) have been fused to TALEN (Box 1) and dCas9 (Gao et al., 2014; Kearns et al., 2015). Additionally, dCas9 has been fused to the catalytic domain of the HAT p300 (Table 1) (Hilton et al., 2015). Although these approaches showed different efficiencies, they indicate the potential use of combining dCas9 with epigenetic enzymes for further investigations of the epigenetic status of any risk-associated SNPs in organoids.

Combining kidney organoids exposed to environmental conditions that mimic disease-related contexts (i.e. high glucose, AGEs, hypoxia) with the constantly evolving genome editing toolbox may allow us to perform site-specific recruitment of engineered chromatin regulators to modulate epigenetic marks at DNA regulatory elements under these experimental conditions. These approaches could improve our understanding of the function of the epigenetic marks that underlie kidney development and disease. Moreover, the combination of genome and epigenome editing tools in the organoid setting could help uncover novel molecular pathways associated with the genotypes detected by global analyses, including GWAS and EWAS, by elucidating genotype-epigenotype interactions.

## Conclusions

In the past decades, several studies have demonstrated the impact of epigenetic modifications on renal disease and its progression towards CKD and ESRD, improving our understanding of these conditions. However, the development of innovative approaches for diagnosis, prevention and treatment of CKD represents a major challenge for the nephrology community. The emergence of GWAS has greatly expanded our knowledge of risk alleles related to CKD and ESRD. However, for most of the reported CKD-associated loci, the effects on disease pathogenesis remain unknown. Recently, researchers have shown that it is possible to recapitulate the development of renal cells from undifferentiated hPSCs derived from human embryos and from patients (iPSCs). Kidney organoids are an unprecedented *in vitro* cellular model that can help to unravel the molecular mechanisms driving kidney differentiation and, importantly, disease. The organoid technology represents a powerful tool that can pave the way towards the development of high-throughput systems for the screening of genetic variants, epigenetic modifications or changes in chromatin structure that drive renal disease. Kidney organoids may also help researchers bridge the translational gap for the implementation of genetic and epigenetic therapies into the clinic. Altogether, we hope that the next few years will bring us closer to a new era of knowledge, taking advantage of the application of genome editing tools and stem cell bioengineering for the identification of novel therapeutic strategies targeting the renal epigenome during disease.
